# Fine Epitope Mapping of the Central Immunodominant Region of Nucleoprotein from Crimean-Congo Hemorrhagic Fever Virus (CCHFV)

**DOI:** 10.1371/journal.pone.0108419

**Published:** 2014-11-03

**Authors:** Dongliang Liu, Yang Li, Jing Zhao, Fei Deng, Xiaomei Duan, Chun Kou, Ting Wu, Yijie Li, Yongxing Wang, Ji Ma, Jianhua Yang, Zhihong Hu, Fuchun Zhang, Yujiang Zhang, Surong Sun

**Affiliations:** 1 Xinjiang Key Laboratory of Biological Resources and Genetic Engineering, College of Life Science and Technology, Xinjiang University, Urumqi, Xinjiang, China; 2 State Key Laboratory of Virology, Chinese Academy of Sciences, Wuhan, Hubei, China; 3 Center for Disease Control and Prevention of the Xinjiang Uyghur Autonomous Region, Urumqi, Xinjiang, China; 4 Texas Children's Cancer Center, Department of Pediatrics, Dan L. Duncan Cancer Center, Baylor College of Medicine, Houston, Texas, United States of America; Division of Clinical Research, United States of America

## Abstract

Crimean-Congo hemorrhagic fever (CCHF), a severe viral disease known to have occurred in over 30 countries and distinct regions, is caused by the tick-borne CCHF virus (CCHFV). Nucleocapsid protein (NP), which is encoded by the S gene, is the primary antigen detectable in infected cells. The goal of the present study was to map the minimal motifs of B-cell epitopes (BCEs) on NP. Five precise BCEs (E1, ^247^FDEAKK^252^; E2a, ^254^VEAL^257^; E2b, ^258^NGYLNKH^264^; E3, ^267^EVDKA^271^; and E4, ^274^DSMITN^279^) identified through the use of rabbit antiserum, and one BCE (E5, ^258^NGYL^261^) recognized using a mouse monoclonal antibody, were confirmed to be within the central region of NP and were partially represented among the predicted epitopes. Notably, the five BCEs identified using the rabbit sera were able to react with positive serum mixtures from five sheep which had been infected naturally with CCHFV. The multiple sequence alignment (MSA) revealed high conservation of the identified BCEs among ten CCHFV strains from different areas. Interestingly, the identified BCEs with only one residue variation can apparently be recognized by the positive sera of sheep naturally infected with CCHFV. Computer-generated three-dimensional structural models indicated that all the antigenic motifs are located on the surface of the NP stalk domain. This report represents the first identification and mapping of the minimal BCEs of CCHFV-NP along with an analysis of their primary and structural properties. Our identification of the minimal linear BCEs of CCHFV-NP may provide fundamental data for developing rapid diagnostic reagents and illuminating the pathogenic mechanism of CCHFV.

## Introduction

The Crimean-Congo hemorrhagic fever virus (CCHFV) is a human pathogenic agent that causes Crimean-Congo hemorrhagic fever (CCHF), a severe disease with case-fatality rates up to 30% [1]–[3]. CCHFV is broadly distributed across much of the Middle East, Africa, and Asia as well and has also been found in parts of Eastern Europe [4]–[6]. Humans are generally infected through tick bites, direct contact with blood or tissue of infected livestock, or through nosocomial infections [7]–[9]. In China, the first CCHF cases were reported in 1965 when the CCHFV strain BA66019 was isolated in a patient living in Bachu County of the Xinjiang Autonomous Region, which is now known to have the highest occurrences of CCHF in the country [10]. Despite the high mortality associated with CCHF, the biology and pathogenesis of the disease remain poorly understood for several reasons: CCHF outbreaks are sporadic and have been generally restricted to a relatively small number of cases, limited animal model development, and the handling of the infectious virus requires the highest level of laboratory containment (BSL-4) [11]. Thus, early diagnosis and vaccine development are critical for both patient survival and for the prevention of potential nosocomial infection and transmission in China.

CCHFV belongs to the *Nairovirus* genus within the family Bunyaviridae [2], [12]. The genome consists of three negative-stranded RNAs, designated as small (S), medium (M) and large (L) in accordance with their relative nucleotide length, and which encode the viral nucleocapsid protein (NP), the glycoprotein precursor (GP) and the putative RNA-dependent polymerase, respectively [13]. Studies have indicated that NP is the predominant protein which is present in high levels early after infection, thereby inducing a high immune response that can be detected in infected cells [14]-[17]. As a major protein primarily detected during the viral invasion phase, NP has been increasingly regarded as an important target of antivirus and clinical diagnosis [2]. In previous studies, complete NP expressed in bacteria has been used to detect CCHFV immunoglobulin G (IgG) and IgM antibodies; however, the instability of the protein has limited its application for routine use [18]–[20]. Thus there is a need to develop truncated NP or a multi-epitope peptide for CCHF diagnosis. In a prior study, Saijo et al. [21] reported that high titer sera of CCHF patients reacted only with amino acid residues 201 to 306 (NP^201-306^) of the NP central fragment, a highly conserved region among various isolates. In our previous study, the NP region containing amino acid residues 237 to 305 (NP^237-305^) was found to have remarkable reactivity both with a rabbit polyclonal antibody (pAb) against CCHFV-NP and with a mouse monoclonal antibody (mAb) 14B7 in Western blotting analysis [22].

Advances have made epitope mapping much easier today than it was before. Many approaches and technologies, including recombinant DNA [23], peptide synthesis [24], and peptide [25] or protein display [26] have highlighted the need for epitope mapping and raised the possibility of mapping to a sufficient level the epitopes of certain antigens of interest [27]. Biosynthetic peptide technology is often used to express several 15–25mer peptide segments covering a certain target protein to determine the presence of an antigenic region or regions for a mAb or pAb by the use of Western blotting. Epitope mapping can be subsequently performed with a set of synthetic overlapping 8mer peptides for the positive segment(s) detected by immunoblotting [28]–[30]. Herein, based on the findings of a previous study, we describe the fine epitope mapping of immunodominant region NP^237−305^ of the CCHFV using an improved biosynthetic peptide method [28], [29].

In this paper, a total of six overlapping 16–22mer peptides (Y1–Y6) and forty-one 8mer peptides (P1–P41), both fused with a truncated carrier protein, were biosynthesized and expressed for minimal epitope mapping of the antigenic properties of NP^237−305^. Five potential pAb BCEs and one potential mAb BCE were identified and mapped on the stalk region of CCHFV-NP for the first time.

## Materials and Methods

### Ethics Statement

The study was approved by the Research Ethics Committee (Animal Ethics Committee of Xinjiang University) and the procedures that followed were in accordance with the policies and regulations of experimental animals of China. The field studies in Bachu County were permitted by Xinjiang Wildlife Conservation Association (XJWCA). The serum samples were collected using method of random sampling and this process was not involving sacrifice.

### Plasmids, Antibodies and Strains

The plasmids pGEX-KG and pXXGST-1 [28] were used to express biosynthetic peptides. The prokaryotic expression plasmid pGEX-KG was maintained by the Xinjiang Key Laboratory of Biological Resources and Genetic Engineering, and pXXGST-1 was donated by Professor Wanxiang Xu of the Shanghai Institute of Planned Parenthood Research. Rabbit polyclonal antibody against CCHFV-NP (pAb) was prepared as previously described [31]. A mouse monoclonal antibody cell line (14B7) secreting IgM type monoclonal antibody 14B7 against CCHFV was obtained from Xinjiang Centers for Disease Control and Prevention (XJCDC). A high titer of mAb was separated from mice ascites [32]. A pooled sheep serum of five samples collected from Bachu County with a confirmed history of CCHFV infection was included in the study and used for reconfirming the antigenicity of identified BCEs of CCHFV-NP in Western blotting assay. Serum sample of one healthy sheep with no history of CCHFV infection was used as negative control. All the sheep sera used in the study were collected in 2005 and kindly provided by Professor Zhang Yujiang of XJCDC [33]. The serum samples of sheep infected with CCHFV were previously identified by using indirect immunofluorescent assay (IFA) and reverse transcription polymerase chain reaction (RT-PCR) [33]. *Escherichia coli* (*E. coli*) BL21 (DE3) competent cells, used for expression of recombinant plasmids, were purchased from Beijing TransGen Biotech Co., Ltd.

### Epitope Prediction

To predict the B-cell epitopes (BCEs) on the NP^237−305^ fragment of CCHFV, the corresponding amino acid sequence was analyzed using the DNAStar Protean system. Secondary structure prediction of the truncated protein was performed by using the methods of Garnier and Robson [34] and Chou and Fasman [35]. The surface properties of the structural proteins, namely, hydrophilicity, flexibility, accessibility and antigenicity, were analyzed using the methods of Kyte and Doolittle [36], Karplus and Schulz [37], Emini [38] and Jameson and Wolf [39], respectively. According to the results obtained using these methods, peptides with good hydrophilicity, high accessibility, high flexibility and strong antigenicity were selected as epitope candidates. In general, peptides located in α-spiral and β-sheet regions, which do not readily form epitope regions, were excluded [40].

### Biosynthesis of 8-22mer Peptides and Recombinant Plasmid Construction

**Figure 1:**
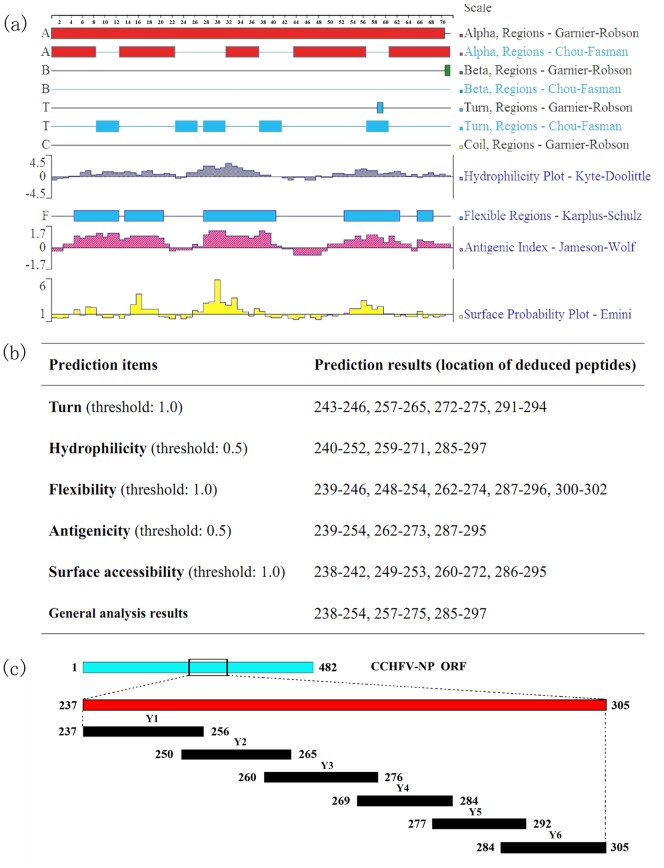
Prediction and mapping strategy of the epitopes in the central region of CCHFV-NP. (**a**) Epitope prediction for amino acid residues 237–305 of the NP sequence of the YL04057 strain using DNAStar Protean software. The secondary structure, flexibility plot, hydrophilicity, surface probability, and antigenicity index for NP^237−305^ were taken into consideration. (**b**) Epitope predictions of the NP^237−305^ fragment of the YL04057 strain based on various principles. (**c**) Schematic of the BCE mapping strategy with six 16–22mer overlapping biosynthetic peptides spanning the NP^237−305^ fragment. The blue band represents the full length nucleoprotein. The red band represents the immunodominant fragments of YL04057 NP.

Six biosynthetic 16–22mer peptides (designated Y1–Y6) spanning the NP^237−305^ segment and overlapping 6 ∼ 9 amino acid residues each other, which all fused with GST or a truncated GST188 carrier protein were expressed in *E. coli*, respectively [30]. The sequences of the peptides were as follows: Y1 (KLAETEGKGVFDEAKKTVEA), Y2 (AKKTVEALNGYLNKHK), Y3 (YLNKHKDEVDKASADSM), Y4 (DKASADSMITNLLKHI), Y5 (ITNLLKHIAKAQELYK) and Y6 (IAKAQELYKNSSALRAQGAQID), which correspond to NP^237−256^, NP^250−265^, NP^260−276^, NP^269−284^, NP^277−292^ and NP^284−305^, respectively. For the immunodominant peptides identified (Y1–Y4), four additional sets of 8mer peptides spanning the Y1 to Y4 fragments overlapping 7 residues each other were generated (totaling 41 biosynthetic peptides designated P1 to P41) for fine epitope mapping (Fig. 1c). Briefly, the synthesized DNA fragments encoding the Y1–Y6 and P1–P41 peptides (based on the S gene sequence [41] were flanked by *Bam*H I and TTA-*Sal* I sites at the 5′ and 3′ ends, respectively, then inserted into the *Bam*H I and *Sal* I sites downstream of the GST or GST188 encoding gene in the pGEX-KG or pXXGST-1 plasmid.

### Expression of Fusion Proteins

The resultant recombinant plasmids expressing each 8–22mer peptide fused with GST or GST188 were transformed into *E. coli* BL21 (DE3) competent cells. Each recombinant clone was cultivated in 3 mL LB medium containing 100 µg/mL ampicillin at 30°C with continuous shaking at 200 rpm overnight. The next day, 30 µL of cell suspension was added to 3 mL fresh LB medium and grown for 4 h until reaching a bacterial density of 0.6–0.8 at OD600. The cells were grown for an additional 4 h with 0.8 mM IPTG (Y1, Y3 and Y6 fusion peptides) or without IPTG (all other fusion peptides) at 42°C to induce the expression of the recombinant proteins. For the screening of positive recombinant clones, an SDS-PAGE gel was run for each harvested cell pellet, with the pellet corresponding to GST or GST188 protein expressed by pGEX-KG or pXXGST-1 as a negative control. All recombinant clones were subsequently sent out for sequencing determination. The cell pellets containing the short peptide fusion proteins were stored at −20°C.

### SDS-PAGE and Western Blotting

The cell pellets obtained from 3 mL medium were boiled in 400 µL of 1×SDS-PAGE loading buffer for 10 min, and the proteins were resolved by SDS-PAGE under reducing conditions using 15% gels [42]. Gels were either stained with Coomassie brilliant blue R-250 to analyze the bands corresponding to the fusion proteins or processed for Western blotting by electrotransfer of the proteins onto a 0.2 µm nitrocellulose membrane (Whatman GmbH, Dossel, Germany) [43]. Complete transfer of proteins was ensured by staining the nitrocellulose membrane with 0.1% (w/v) Ponceau S dye liquor. After cleaning and blocking, the nitrocellulose membrane was subsequently treated with pAb (1∶1000 dilution in PBS containing 0.05% Tween 20 and 1% skim milk powder), mAb 14B7 (1∶500 dilution) or a pooled serum (1∶100 dilution) collected from sheep with confirmed CCHFV infection. A serum sample from a known healthy sheep with no history of CCHFV infection was used as a negative control. Specific antigen-antibody reactions on the membrane were visualized using goat anti-rabbit IgG, goat anti-mouse IgM, or rabbit anti-sheep IgG conjugated to horseradish peroxidase (HRP) (Proteintech Group, Chicago, USA) at a 1∶1000 dilution. The blot was performed using ECL plus Western blotting detection reagent (GE Healthcare, Buckinghamshire, UK) according to the manufacturer's instructions.

### Sequence Conservation Analysis and Three-Dimensional Modeling

To assess the sequence conservation of the identified epitopes, nine NP amino acid sequences collected from strains in different countries were obtained from GenBank. Amino acid residues 170–305 of the NP sequences from the nine virus strains were selected for multiple alignment analysis against the corresponding sequence of the YL04057 strain (GenBank code: ACM78470.1) using the ClustalW program (http://www.ebi.ac.uk/services) [44].

Three-dimensional structures of the immunodominant epitopes identified using pAb and 14B7 were simulated using PyMOL™ software [45].

## Results

### BCE Prediction and Mapping Strategy

Accessibility, variability, fragment mobility, charge distribution and hydrophilicity are important features of antigenic epitopes. The presence of flexible regions, such as coil and turn regions, provide further evidence for epitope identification. In this study, the secondary structure of NP^237−305^ was predicted using the methods of Garnier and Robson [34] and Chou and Fasman [35] and the NP gene sequence of YL04057 CCHFV. A hydrophilicity plot, flexibility plot, surface probability plot and antigenic index for the truncated protein were obtained using the methods of Kyte and Doolittle [36], Karplus and Schulz [37], Emini [38] and Jameson and Wolf [39], respectively (Fig. 1a). The potential BCEs on NP^237−305^ were predicted (Fig. 1b) based on the methods mentioned above. The finding that the secondary structure of the NP^237−305^ fragment consists of five main turn motifs suggested the presence of multiple significant BCEs in this region. In our previous study, the NP^237−305^ truncated fragment was found to exhibit remarkable antigen-antibody reactivity when either pAb or mAb was used in Western blotting analysis [22]. However, other unpredicted amino acids in this same region should also be considered because they may also contain BCEs, some of which may be predominant BCEs. To identify how many epitopes there are in the fragment of NP, we therefore designed a feasible strategy for BCE mapping of the NP^237−305^ (Fig. 1c). Briefly, six truncated polypeptides (Y1–Y6) spanning NP^237−305^ were incorporated into prokaryotic expression plasmids. Based on the results of the Western blotting analysis, sets of 8mer peptides were constructed for each of the immunodominant polypeptides identified for further BCE mapping.

### Mapping Epitopes on CCHFV-NP Using pAb

**Figure 2:**
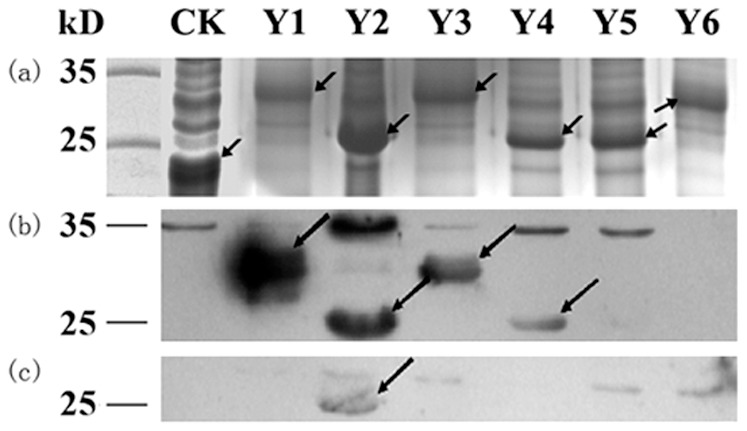
Prokaryotic expression and immunoblotting analysis of Y1–Y6 fused proteins. (**a**) SDS-PAGE analysis of expressed pXXGST-1 (CK) and Y1-Y6 peptides fused with a GST (Y1, Y3 and Y6) or GST188 tag (Y2, Y4 and Y5). (**b**) Western blotting of fusion proteins Y1–Y6 using the rabbit polyclonal antibody against CCHFV-NP. (**c**) Western blotting of fusion proteins Y1–Y6 using the mouse IgM-type monoclonal antibody 14B7 against CCHFV. The arrows represent expressed target peptides in SDS-PAGE and the corresponding positive antigenic-peptides in Western Blotting analysis.

All 16–22mer Peptides fused with a GST or GST188 carrier were expressed through constructing short peptide fusion expression plasmids using each synthesized encoding DNA fragments [28]. To define the fine epitopes on the NP^237−305^ fragment of CCHFV, epitope mapping was performed in two steps. For the first round of antigenic peptide mapping, NP^237−305^ was divided into six overlapping fragments (Y1–Y6), which were fused with GST/GST188 and expressed in *E. coli*, respectively. As determined by SDS-PAGE, bands corresponding to the GST-fused proteins (Lane 2, 4 and 7) were approximately 33 kD, and those corresponding to the GST188-fused proteins (Lane 3, 5 and 6) were approximately 25 kD (Fig. 2a). Western blot analysis showed that pAb reacted with polypeptides Y1–Y4 (Fig. 2b).

**Figure 3:**
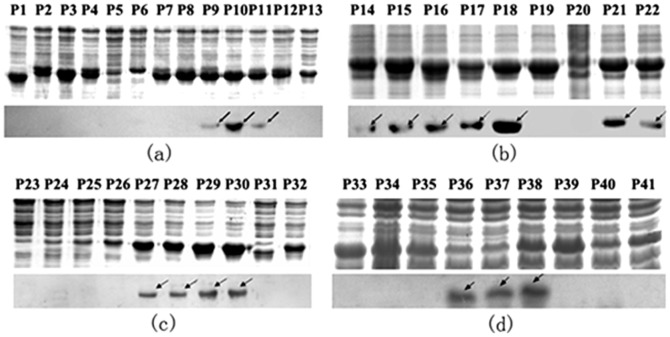
SDS-PAGE identification and Western blotting analysis of the minimal epitopes on NP^237−305^ using pAb. (**a**) Thirteen 8mer peptides (P1–P13) corresponding to the Y1 protein. (**b**) Nine 8mer peptides (P14–P22) corresponding to the Y2 protein. (**c**) Ten 8mer peptides (P23–P32) corresponding to the Y3 protein. (**d**) Nine 8mer peptides (P33–P41) corresponding to the Y4 protein. The arrows stand for 8mer peptides which display a positive antigen-antibody reaction in Western Blotting analysis.

**Figure 4:**
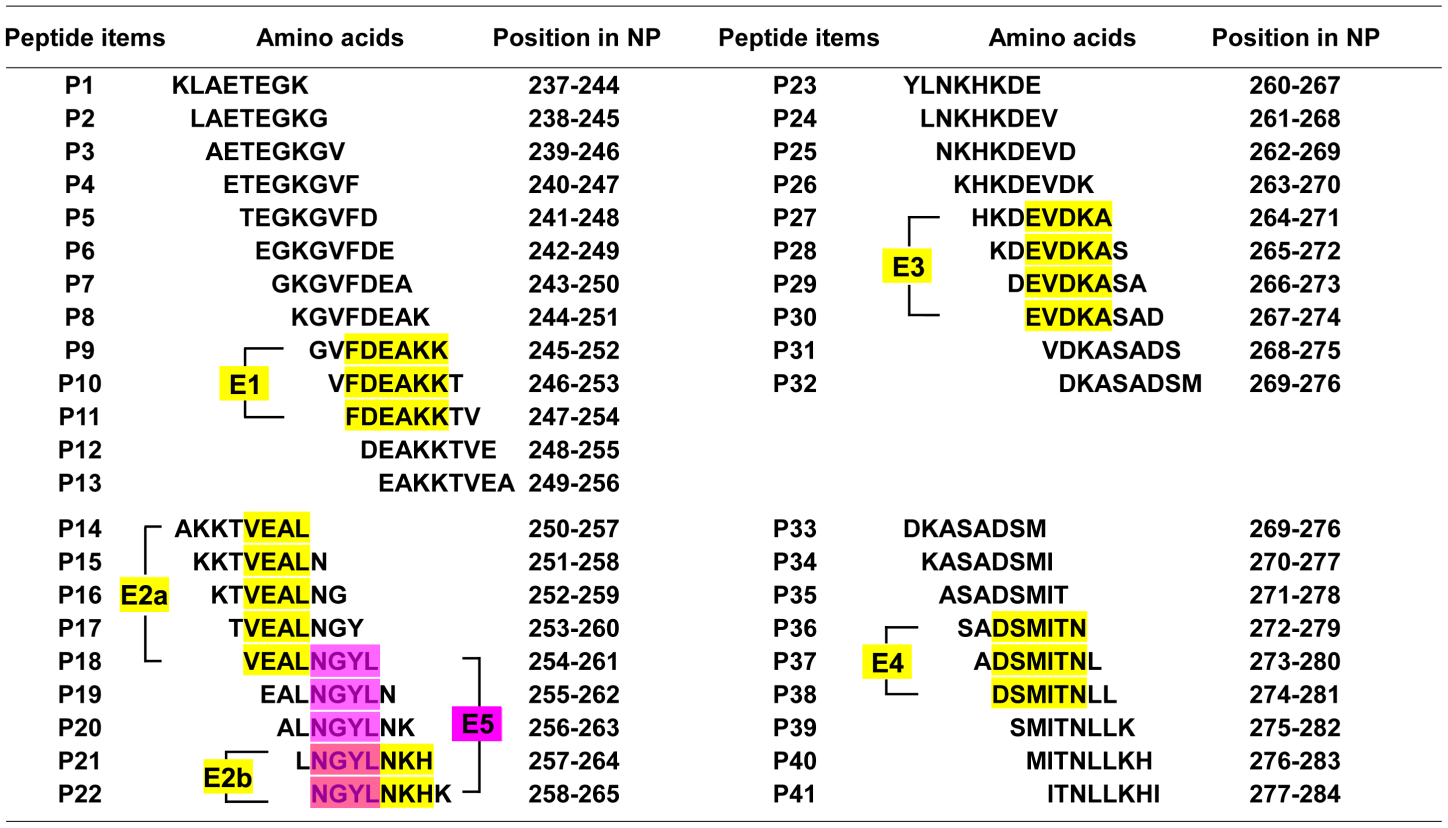
The synthetic 8mer peptide sequences derived from a span of the immunodominant peptides Y1, Y2, Y3, and Y4 respectively. The yellow and magenta highlighting represents the common sequences among peptides which react with pAb or mAb using Western blotting analysis.

To further map the epitopes on NP^237−305^, four sets of 8mer peptides spanning Y1 to Y4 were constructed, which have an overlap of seven amino acid residues each other in second round of fine epitope mapping. A total of 41 recombinant 8mer peptides (designated P1 to P41) were constructed and expressed in *E. coli* (Fig. 3). Among the 13 recombinant clones corresponding to Y1 (Fig. 3a), Western blot analysis showed that 8mer peptides P9 (GVFDEAKK), P10 (VFDEAKKT) and P11 (FDEAKKTV) were recognized by pAb against NP, suggesting that the epitope minimal motif within Y1 was the FDEAKK (named as epitope 1, E1) according to their shared residues number (Fig. 4). Three antigenic peptides Y2–Y4 were similarly identified and analyzed (Fig. 3 and 4): the fine epitopes were the VEAL (E2a) and NGYLNKH (E2b) in Y2, EVDKA (E3) in Y3 and DSMITN (E4) in Y4, respectively. Thus, five specific BCE motifs within the NP^237−305^ segment were found using rabbit pAb to NP.

### Epitope Mapping Using mAb 14B7 Against CCHFV-NP

**Figure 5:**
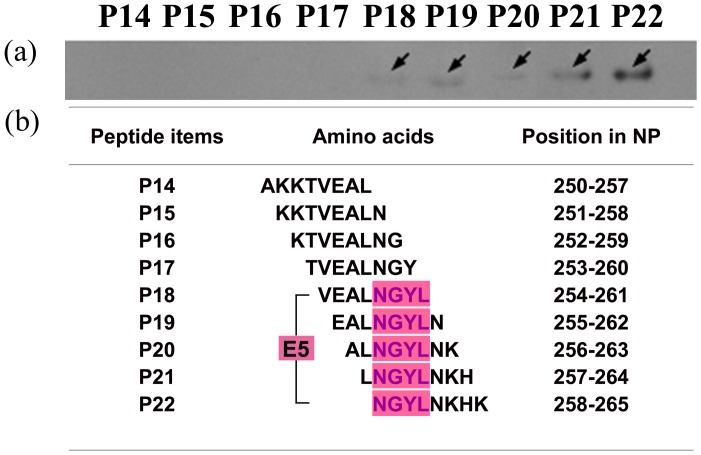
Minimal epitope identification on CCHFV-NP using mAb 14B7. (**a**) A reactivity profile of the 8mer peptides P14–P22 corresponding to Y2 using Western blotting analysis. (**b**) Sequences of the 8mer peptides and their positions in NP. Magenta highlighting indicates the common sequence identified as the minimal BCE (E5) on NP using 14B7. The arrows represent 8mer peptides which display positive antigen-antibody reactions using Western Blotting analysis.

In our previous study, NP^237−305^ was also found to exhibit antigen-antibody reactivity with 14B7. To reveal its antibody-reactive epitope motif, using same strategy described above to map its fine epitope motif. That is, the mAb 14B7 was identified to recognize antigenic peptide Y2 in the first round of mapping (Fig. 2c) and then its epitope motif was confirm as NGYL (designated as E5, amino acid residues 258–261) in the second round of fine mapping (Fig. 5). Interestingly, its epitope motif was located in the E2b identified by the rabbit pAb, suggesting the diversity of antibody production in mouse and rabbit.

### Determination of the Antigenicity of Identified BCEs by CCHFV Antibody-positive Sheep Sera

**Figure 6:**
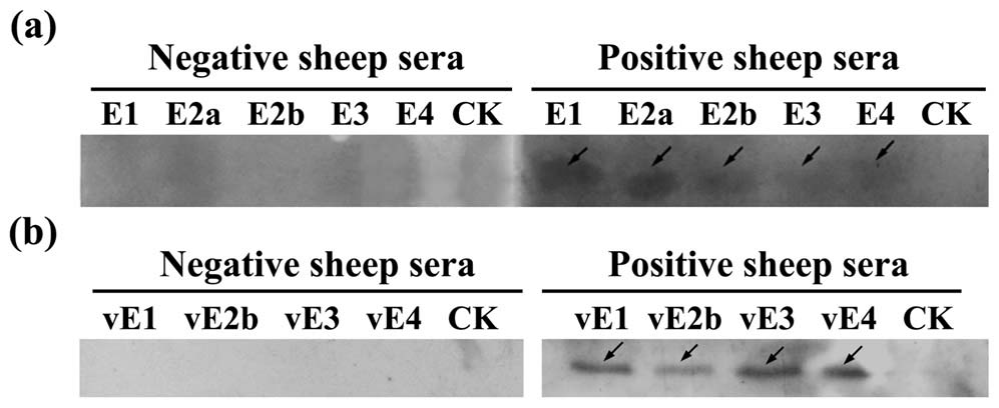
Western blot of five 8-mer peptides containing identified BCEs with or without one residue variation performed using positive sheep sera with a confirmed history of CCHFV infection. Five randomly selected 8-mer peptides containing identified BCEs (**a**) or BCEs with one residue variation (**b**) expressed as GST188 fusion protein in *E. coli*. A serum sample of healthy sheep with no history of CCHFV infection was used as a negative control. CK was a GST188 protein tag. The arrows represent 8mer peptides displaying positive antigen-antibody reactions based upon Western Blotting analysis.

To determine whether the BCEs identified are rabbit/mouse specific or also recognizable by the immune systems of other host species, five randomly selected 8-mer peptides, each of which containing one of the five pAb-identified BCEs, were carried out Western blot test by using sheep sera with or without CCHFV infection (Fig. 6a). As shown in this study, the serum samples of sheep with a confirmed history of CCHFV infection could react with all the five 8-mer peptides to varying degrees, while the CCHFV antibody-negative sera could not react with anyone of them. Of the five peptides, P10 (containing E1) and P18 (containing E2a) showed the strongest antigen-antibody reaction activities with CCHFV-infected sheep sera; Meanwhile, P29 (containing E3) and P38 (containing E4) displayed the weakest reaction intensity among the five 8-mer peptides.

### Sequence Conservation Analysis and Three-Dimensional Modeling

**Figure 7:**
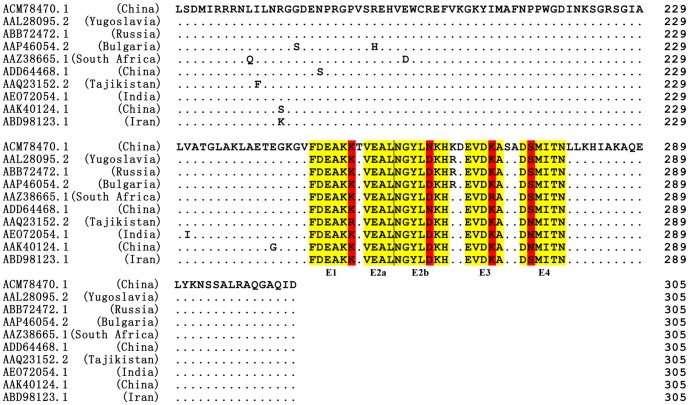
Amino acid sequence comparison of the NP^170−305^ fragment from the YL04057 strain (ACM78470. 1) and other CCHFV strains using the ClustalW program. The GenBank codes and sources are shown at left. The five minimal epitopes E1, E2a, E2b, E3, and E4 recognized by pAb are highlighted in yellow, and the variable amino acids within the minimal epitopes are highlighted in red. Dots (.) indicate identical amino acids within the ten strains.

To analyze primary structural properties of identified each BCE, the sequence corresponding to amino acid residues 170–305, which contains the identified BCEs and flanking sequences, was used to conduct multiple sequence alignment (MSA) (Fig. 7). The analysis revealed that this region (using the sequence from Chinese strain YL04057, ACM78470.1) is highly conserved when compared to the corresponding region from other CCHFV strains, with 90.4% sequence identity. Notably, an even higher sequence identity (92.8%) was found for NP^237−305^ compared to the other nine strains. The five BCEs (E5 epitope motif was included within E2b) identified were also found to be highly conserved. Only single amino acid differences were found for four BCEs (E1, E2b, E3 and E4) compared with the other strains. To further determine whether the epitope peptides with single residue difference revealed in Fig. 7 could be used as a or all universal diagnostic reagent(s), we prepared four biosynthetic peptides (vE1, FDEAKR; vE2b, NGYLDKH; vE3, EVDRA; vE4, DNMITN) using methods mentioned above and explored their antigenic properties. Specifically, a single amino acid substitution was made within E1 (K252R), E2b (N262D), E3 (K270R), and E4 (S275N). Our investigation demonstrated that the four biosynthetic peptides with one variable residue (vE1, vE2a, vE3, vE4) can remarkably react with CCHFV antibody-positive sheep sera compared with the negative sheep sera panel (Fig. 6b), suggesting that BCEs E1–E4 derived from different CCHFV strains shared conservation in antigenicity aspect. Intriguingly, we found the E2a epitope (VEAL) and E5 epitope (NGYL) identified using mAb 14B7 were highly conserved in a majority of CCHFV isolates (Fig. 7).

**Figure 8:**
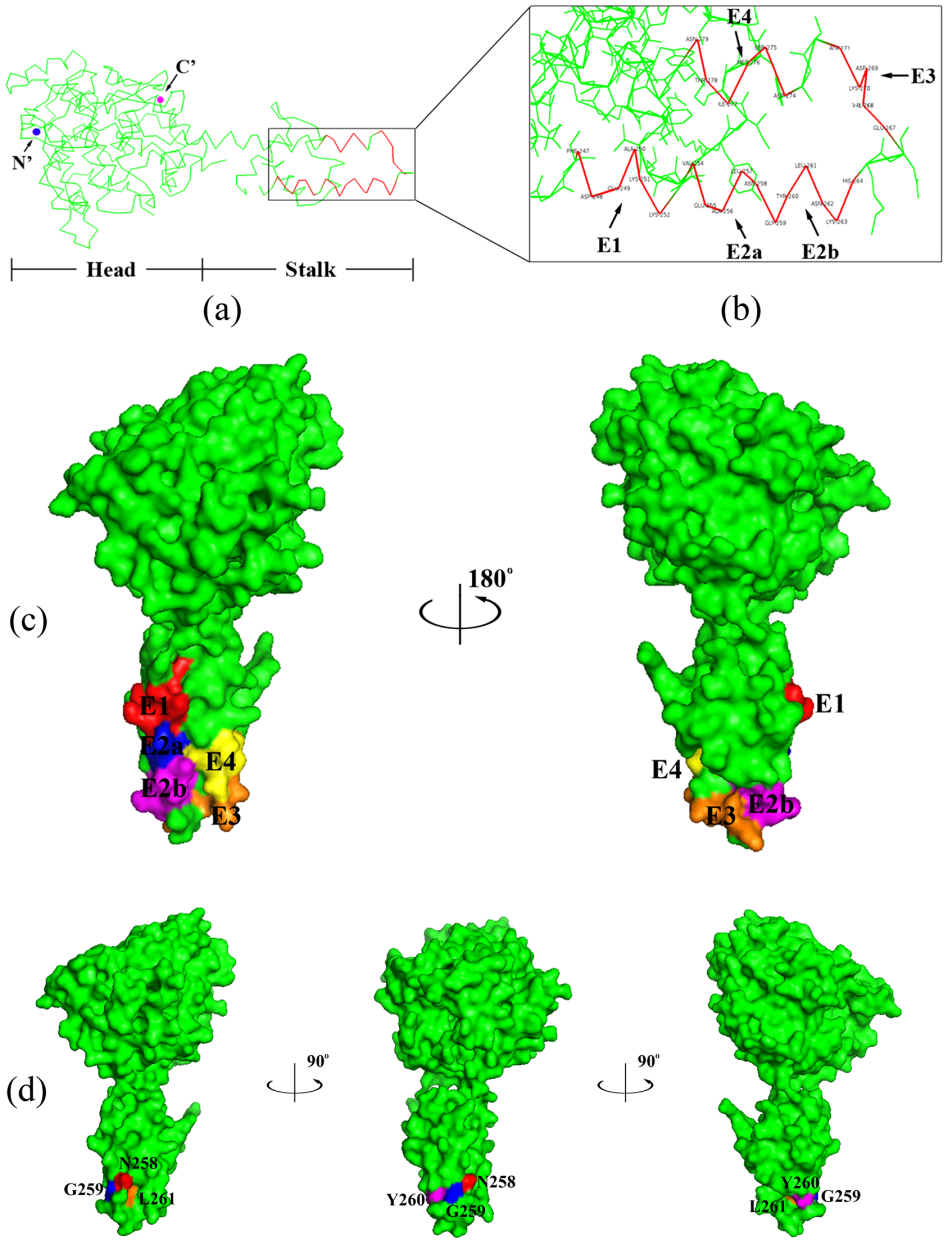
Location and three-dimensional structure of the epitopes identified using pAb and mAb 14B7 on CCHFV-NP. (**a**) The ribbon diagram shows the overall secondary structure of CCHFV-NP from strain YL04057 (PDB code: 3U3I). The motifs within the frame indicate the five minimal epitopes E1–E4. (**b**) E1–E4 sites on the CCHFV-NP stalk domain. (**c**) Surface properties of CCHFV-NP. The molecular surfaces of E1 (red), E2a (blue), E2b (magenta), E3 (orange) and E4 (yellow) are shown. (**d**) The structural representations show the location and spatial conformation of epitope E5 (tetrapeptide “NGYL”) identified by Amb 14B7. Residues N258, G259, Y260, and L261 are shown in different colors. The figures were generated using the PyMOL molecular graphics system.

Computer modeling using PyMOL™ software indicated that all of the antigenic motifs are located on the stalk domain of CCHFV-NP (Fig. 8a). Furthermore, the five BCEs are located in a flexible “helix-turn-helix” (HTH) structure (Fig. 8b). According to the surface representations (Fig. 8c and 8d), all of the identified BCEs are located on the surface of NP, which is consistent with the antigenic principles of surface accessibility and hydrophilicity.

## Discussion

Nucleocapsid research is an important branch of viral study, as virus nucleocapsids may stimulate human immune responses, most of which are of the humoral immunity type [46]. Thus, the identification and mapping of minimal BCEs on NP represent significant steps in the development of novel diagnostic tools and multi-epitope peptide vaccines. In a previous study, a bacterially expressed recombinant NP antigen was used to detect IgG antibodies against CCHFV; the instability however, of the protein in soluble expression as well as serological diagnosis restricted the application of this protein [47], [48]. The use of non-complete NP or multi-epitope peptides for CCHF diagnosis has attracted increasing attention, along with CCHF studies in general. At the same time, there have been increased efforts related to the epitope mapping of CCHFV-NP. For instance, Saijo et al. reported that in Western blotting analysis, high titer sera of CCHF patients reacted only with the highly conserved NP fragment which contained the amino acid residues 201 to 306 (NP^201−306^) [21]. Similarly, Burt et al. found that NP^123−396^ of CCHFV includes a highly antigenic region with application toward the development of antibody detection assays [48]. Previously, our group showed that NP^237−305^ is an immunogenic region of CCHFV-NP using a polyclonal antibody and two monoclonal antibodies against CCHFV with Western blot analysis [22]. It is worth noting that the NP^237−305^ region is smaller and more detailed and completely encompassed by the NP^201−306^ and NP^123−396^ regions. The consistent finding from these independent research groups suggests that the high antigenicity region of CCHFV-NP is located in the central region of NP rather than the N- or C-terminal regions. Although several antigenic peptides have been mapped on CCHFV-NP, to our knowledge, no minimal motifs have been previously identified, due to methodological limitations.

The biosynthetic peptide method has been successfully used by several research groups to identify the minimal epitopes on human zona pellucida protein [28]–[30]. In the present study, we used two prokaryotic plasmids for expressing 8–22mer peptides fused with a GST or GST188 tag to avoid the influence of different expression systems on the stability and antigenicity of the recombinant peptides generated. The simplicity, cost effectiveness, reliability, and adaptability of this approach are highly suitable for minimal motif identification [28]. Herein, we demonstrate the use of this method in mapping the minimal motifs of the BCEs of CCHFV-NP. Thus, this methodology may accelerate research requiring the minimal motif mapping of known viral antigenic epitope fragments. In the present study, we mapped six minimal BCEs on NP, five of which were identified by pAb (E1, ^247^FDEAKK^252^; E2a, ^254^VEAL^257^; E2b, ^258^NGYLNKH^264^; E3, ^267^EVDKA^271^; and E4, ^274^DSMITN^279^) and one by mAb (E5, ^258^NGYL^261^). Herein, the antigenicity of the five pAb-identified BCEs was reconfirmed by utilizing natural sera from the sheep with CCHFV infection history, indicating that the identified BCEs may have significant potential in acting as a diagnostic tool to identify whether certain wild animals or even human beings were infected by CCHFV in natural conditions. Additionally, four of the six BCEs were identified in our antigen prediction analysis (Fig. 1b), demonstrating that the epitope prediction tool combined with the biosynthetic peptide method is a reliable approach for epitope mapping and may reduce the experimental effort and expense of identifying and mapping epitopes for immunodiagnostics.

The five minimal BCEs found on NP^237−305^ span amino acid residues 247 to 279 of CCHFV-NP, and all of them were found to have high sequence similarity among different CCHFV strains according to MSA analysis (88.57% for E2b, 95% for E4, 98% for E3, 98.33% for E1, and 100% for E2a and E5) (Fig. 7). To give specifics, the lysine252 was replaced by arginine within E1 (K252R) in one strain. In certain strains, a single amino acid substitution was also found within E2b (N262D), E3 (K270R), and E4 (S275N). Despite one residue difference, the antigen-antibody reaction was still obvious when using positive sera of sheep naturally infected with CCHFV (Fig. 6b), reflecting highly antigenic conservation. However, ideally, the sera of CCHF patients should be utilized to verify the conservation and specificity of BCEs, which is crucial for future applications in CCHF diagnosis and prevention. In this study, we only provided the fundamental data that the antigenicity of CCHFV-NP was researched using rabbit polyclonal antiserum against the CCHFV-NP, using the mouse monoclonal antibody against the CCHFV, and using the sera of sheep naturally infected with CCHFV. Thereby, the properties, structure, antigenicity, and immunogenicity of the NP protein, and in particular, identification of human sera infected with CCHFV will be further studied in order to be applied to CCHF diagnosis and therapy in the future. To our knowledge, the ten CCHFV strains used were isolated in countries which were directly affected by at least one of the five CCHF-epidemic areas, namely countries in Europe, Africa, Central Asia, South Asia, and the Middle East (Table S1). As depicted, epitopes E2a and E5 showed 100% conservative properties among all strains from the five CCHF-epidemic areas. Epitope E1 was fully conserved in the countries of Europe, Africa, South Asia, the Middle East, and part of Central Asia. Similarly, epitope E3 also displayed complete homology in the different CCHF-epidemic areas, with the exception of Africa. It is worth noting that the epitope E2b from isolate YL04057, though it was not consistent with the other eight strains from different CCHF-epidemic areas, had merely one amino acid difference among ten CCHFV strains. As far as we know, eleven complete NP sequences of CCHFV strains isolated within China have been registered in the GenBank database. To further confirm whether the epitope E2b (^258^NGYLNKH^264^) showed high homology among strains from China, the sequences of the eleven Chinese strains corresponding to amino acid residues 241 to 300 of NP were retrieved from the GenBank for sequence alignment using the ClustalW program (Figure S1). Our study indicated that there was only a single difference, which of asparagine changing to aspartic acid (N262D) within epitope E2b in four strains of the eleven, suggesting that asparagine262 may well exist only in the Chinese CCHFV strains.

It has been previously reported that the Dugbe virus, another member of the *Nairovirus* genus, shares some antigenic and genetic properties with CCHFV [49]. Based on our findings, however, the amino acid sequences of NP^247-279^ of the two viruses (GenBank codes: ACM78470.1 and AAL73396.1) display only a 9.1% similarity, despite a 57.4% sequence similarity between the two complete NP sequences (data not given). These findings suggest that the identified BCEs may be unique to CCHFV and thus highly species specific.

To further investigate the structural aspects of the minimal BCEs, the three-dimensional structure of YL04057 NP was retrieved from the Protein Databank (PDB code: 3U3I). The five pAb BCEs and one mAb BCE were all found to be located on the NP stalk domain (BCE surface properties shown in different colors in Fig. 8c and 8d). Structural analyses, particularly of the surface structure of epitopes, provide a good foundation in the search for and creation of structurally complementary drugs with clinical applications. The flexible “helix-turn-helix” structure containing the five BCEs may form a discontinuous epitope and easily react with antibodies or drugs. Human MxA protein has been shown to inhibit the CCHFV replication process [50]. In a protein-protein docking study of MxA with CCHFV-NP emphasizing epitope-based immunoinformatics, Srinivasan et al. [51] showed a complementary wrapping of the NP stalk around the MxA model. Together with the present findings, these results suggest that the CCHFV-NP stalk domain may play a critical role in immune system processes and virus interaction. Intrabodies, or intracellular antibodies, are powerful tools for cell biology studies as well as therapeutic applications [52]. They are commonly used to either block the intracellular antibody target or to image endogenous target dynamics [53], [54]. It is reported that intrabodies induced cell death via activation of the caspase-3-mediated apoptotic pathway [55]. In a related vein, recent structural studies of CCHFV-NP revealed that the amino acid residues DEVD at positions 266 to 269 on the NP stalk domain comprise a putative cleavage site of caspase-3, indicating that caspase-3 cleavage of NP may represent a host defense mechanism against lytic CCHFV infection [56]–[59]. Interestingly, the occurrence of the DEVD motif is within the epitope-rich region of amino acid residues 247 to 279. Our study also raises the possibility of a combination of caspase-3-dependent apoptosis and intrabody therapy in fighting a CCHFV infection in the future.

In the present study, we identified five fine linear BCEs on the stalk region of CCHFV-NP using a peptide biosynthesis strategy, thereby demonstrating the utility of this approach in peptide-based assays aimed at antibody detection. However, it remains a topic of further research whether the antigenic activities, consisting of specificity and sensitivity, can be enhanced by linearly fusing the five BCEs so that they would be more easily and more effectively applied in clinical diagnosis and epidemiological investigation.

## Conclusion

In this study, the five highly conserved or 100% conserved B-cell epitopes E1, E2a, E2b (which fully overlaps E5), E3 and E4 do not only react with a prepared polyclonal antibody, but also with the positive sera of sheep naturally infected with CCHFV. More importantly, the four epitope mutants vE1, vE2b, vE3, and vE4 are distinctly recognizable through the use of naturally infected sheep sera. Our discovery has demonstrated a high antigenic-conservation of these identified minimal epitopes, which might be useable as universal epitopes in CCHF diagnosis. It is of great importance that human sera infected with CCHFV be used to test these identified epitopes in future study. Furthermore, these BCEs were determined to be located on the surface of the NP stalk region, suggesting they very well may play significant roles in the process of interaction with the host immune system, being easily recognized by antibodies. These findings would provide fundamental data for the development of novel diagnostic reagents and the illumination of the pathogenic mechanism of CCHFV.

## Supporting Information

Figure S1 (DOC)

Table S1 (DOC)
