# Supplementary material for: Fine Epitope Mapping of the Central Immunodominant Region of Nucleoprotein from Crimean-Congo Hemorrhagic Fever Virus (CCHFV)
Source: PLoS One. 2014 Nov 3;9(11):e108419. doi: 10.1371/journal.pone.0108419 (PMC4217714; doi:10.1371/journal.pone.0108419)
Supplement: Figure S1 — (DOC) [file pone.0108419.s001.doc]

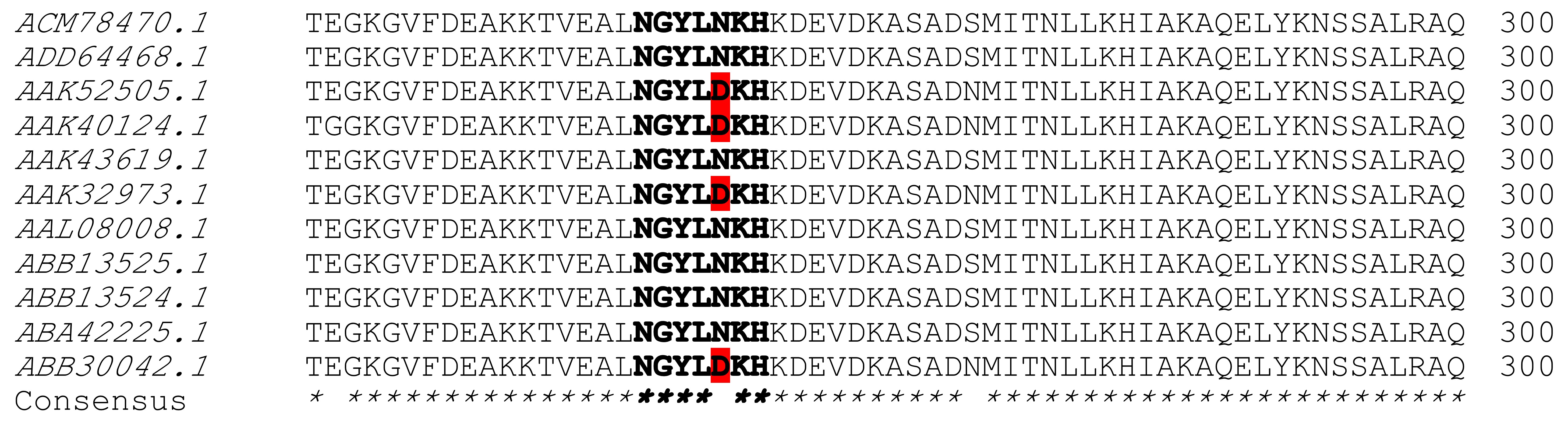


Figure S1. Amino acid conservative analysis of epitope E2b among eleven CCHFV strains isolated from China. As far as we know, eleven complete nucleoprotein (NP) sequences of CCHFV strains isolated from China have been registered in GenBank database. The eleven Chinese strains’ sequences corresponding to amino acid residues 241 to 300 of NP were retrieved from the GenBank for sequence alignment by using ClustalW program. Eleven strains showed good conservative property in E2b site. There was merely a difference (marked with red), [asparagine](app:ds:asparaginate) to aspartic acid (N262D), within epitope E2b in four strains of eleven. Black bold letters (aa 258-264) represent epitope E2b site. Italic letters stand for the GenBank codes of eleven strains. GenBank serial NO. *ACM78470.1* represents the strain YL04057. The asterisk (***) stands for identical amino acid in eleven strains.
