# Supplementary material for: Fine Epitope Mapping of the Central Immunodominant Region of Nucleoprotein from Crimean-Congo Hemorrhagic Fever Virus (CCHFV)
Source: PLoS One. 2014 Nov 3;9(11):e108419. doi: 10.1371/journal.pone.0108419 (PMC4217714; doi:10.1371/journal.pone.0108419)
Supplement: Table S1 — (DOC) [file pone.0108419.s002.doc]

Table S1. Epitope conservative analysis of NP from strain YL04057 with that from other strains in different CCHF-epidemic areas


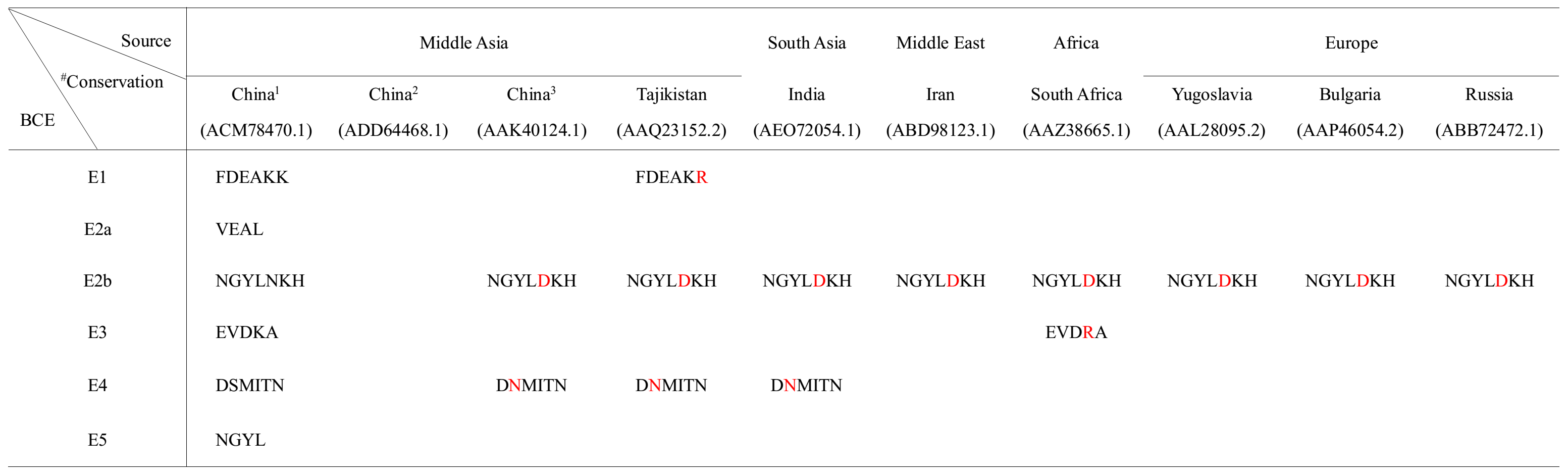


**#:** Blank space represents the fully conservative motif corresponding to identified epitope. Red letter represents the different amino acid compared with identified epitopes of YL04057. The serial numbers in parenthesis represent the GenBank code of CCHFV strains.

1: The strain YL04057.

2, 3: Another two CCHFV strains isolated from Xinjiang Uygur Autonomous Region in China.
